# Supplementary material for: Prevalence of HIV testing uptake among the never-married young men (15–24) in sub-Saharan Africa: An analysis of demographic and health survey data (2015–2020)
Source: PLoS One. 2023 Oct 5;18(10):e0292182. doi: 10.1371/journal.pone.0292182 (PMC10553359; doi:10.1371/journal.pone.0292182)
Supplement: S1 Fig — (DOCX) [file pone.0292182.s001.docx]

**Supplementary file figure 1: Doi plot showing measure of publication bias for the datasets included in the analysis**
