# Supplementary material for: Prevalence of HIV testing uptake among the never-married young men (15–24) in sub-Saharan Africa: An analysis of demographic and health survey data (2015–2020)
Source: PLoS One. 2023 Oct 5;18(10):e0292182. doi: 10.1371/journal.pone.0292182 (PMC10553359; doi:10.1371/journal.pone.0292182)
Supplement: S1 Table — (DOCX) [file pone.0292182.s002.docx]

**Supplementary file Table 1: Sensitivity analysis results**

| **Country** | **Pooled Prevalence** | **LCI 95%** | **HCI 95%** | **Cochran Q** | **p** | **I ^2^** | **I 2 LCI 95%** | **I 2 HCI 95%** |
| --- | --- | --- | --- | --- | --- | --- | --- | --- |
| Angola 2015-16 | 0.33 | 0.23 | 0.43 | 5185.0 | 0.000 | 99.44 | 99.38 | 99.50 |
| Benin 2017-18 | 0.33 | 0.23 | 0.43 | 4968.0 | 0.000 | 99.42 | 99.35 | 99.48 |
| Burundi 2016-17 | 0.32 | 0.22 | 0.42 | 5378.3 | 0.000 | 99.46 | 99.40 | 99.52 |
| Cameroon 2018 | 0.31 | 0.21 | 0.40 | 4802.8 | 0.000 | 99.40 | 99.33 | 99.46 |
| Chad 2014-15 | 0.32 | 0.22 | 0.42 | 5282.5 | 0.000 | 99.45 | 99.39 | 99.51 |
| Ethiopia 2016 | 0.31 | 0.22 | 0.42 | 5361.0 | 0.000 | 99.46 | 99.40 | 99.51 |
| Gambia 2019-20 | 0.32 | 0.22 | 0.42 | 5389.9 | 0.000 | 99.46 | 99.40 | 99.52 |
| Guinea 2018 | 0.33 | 0.23 | 0.43 | 5114.8 | 0.000 | 99.43 | 99.37 | 99.49 |
| Liberia 2019-20 | 0.33 | 0.23 | 0.43 | 5168.0 | 0.000 | 99.44 | 99.37 | 99.50 |
| Malawi 2015-16 | 0.31 | 0.21 | 0.42 | 5325.3 | 0.000 | 99.46 | 99.39 | 99.51 |
| Mali 2018 | 0.32 | 0.23 | 0.42 | 5241.8 | 0.000 | 99.45 | 99.38 | 99.50 |
| Rwanda 2019-20 | 0.31 | 0.22 | 0.42 | 5355.7 | 0.000 | 99.46 | 99.40 | 99.51 |
| Senegal 2019 | 0.33 | 0.23 | 0.43 | 5130.7 | 0.000 | 99.43 | 99.37 | 99.49 |
| Sierra Leone 2019 | 0.31 | 0.22 | 0.41 | 5254.8 | 0.000 | 99.45 | 99.39 | 99.50 |
| South Africa 2016 | 0.31 | 0.21 | 0.41 | 5291.6 | 0.000 | 99.45 | 99.39 | 99.51 |
| Uganda 2016 | 0.31 | 0.21 | 0.41 | 5240.9 | 0.000 | 99.45 | 99.38 | 99.50 |
| Zambia 2018 | 0.29 | 0.20 | 0.39 | 4834.7 | 0.000 | 99.40 | 99.33 | 99.46 |
| Zimbabwe 2015 | 0.31 | 0.21 | 0.42 | 5360.8 | 0.000 | 99.46 | 99.40 | 99.51 |
