# Supplementary material for: Prevalence of HIV testing uptake among the never-married young men (15–24) in sub-Saharan Africa: An analysis of demographic and health survey data (2015–2020)
Source: PLoS One. 2023 Oct 5;18(10):e0292182. doi: 10.1371/journal.pone.0292182 (PMC10553359; doi:10.1371/journal.pone.0292182)
Supplement: Attachment — Submitted filename: Response to Reviewers.docx [file pone.0292182.s003.docx]

The Editor,

PLOS ONE

30^th^ June 2023

**Sub: Response to Decision Letter: Manuscript ID: PONE-D-23-03270: Prevalence of HIV testing among the never-married young men (15-24) in sub-Saharan Africa: an analysis of Demographic and Health Survey data (2015-2022)**

Refer to the above subject matter.

We are grateful to the editor and reviewers for their time and constructive comments on our manuscript. We have taken into consideration the comments and suggestions from reviewers, and we would like to submit a revised version of the manuscript for consideration by the journal. To reflect changes or amendments made in the revised version of the manuscript, changes or amendments have been highlighted in the initial version of the manuscript. In addition, we provide a point-by-point response explaining how we have addressed each of the editors' or reviewers' comments in the section following this one. We are looking forward to hearing the results of your evaluation.

Yours sincerely,

Lead author

Emmanuel Musonda

On behalf of co-authors

**Title: HIV testing rates among the never-married young men in sub-Saharan Africa: an**

**analysis of Demographic and Health Survey data (2010-2020)**

| **Journal Requirements** | | |
| --- | --- | --- |
| **Editors Remarks** | **Authors Response** | **Reference** |
| Thank you for submitting your manuscript to PLOS ONE. After careful consideration, we feel that it has merit but does not fully meet PLOS ONE’s publication criteria as it currently stands. Therefore, we invite you to submit a revised version of the manuscript that addresses the points raised during the review process. | We would like to extend our gratitude to the editor and reviewers for their valuable time and constructive feedback provided on our manuscript. We have diligently worked towards addressing all the raised comments and have incorporated the suggested edits accordingly. We eagerly anticipate any additional support or guidance moving forward. |  |
| When submitting your revision, we need you to address these additional requirements.  1. Please ensure that your manuscript meets PLOS ONE's style requirements, including those for file naming. The PLOS ONE style templates can be found at  https://journals.plos.org/plosone/s/file?id=wjVg/PLOSOne_formatting_sample_main_body.pdf and  https://journals.plos.org/plosone/s/file?id=ba62/PLOSOne_formatting_sample_title_authors_affiliations.pdf | Thank you. We have followed prescribied journal requirement. |  |
| 2. Please upload a copy of Figure 1, to which you refer in your text on page 9. If the figure is no longer to be included as part of the submission please remove all reference to it within the text. | Figure 1 was edited to read Table 1 |  |
| 3. Please review your reference list to ensure that it is complete and correct. If you have cited papers that have been retracted, please include the rationale for doing so in the manuscript text, or remove these references and replace them with relevant current references. Any changes to the reference list should be mentioned in the rebuttal letter that accompanies your revised manuscript. If you need to cite a retracted article, indicate the article’s retracted status in the References list and also include a citation and full reference for the retraction notice. | Thank you. We have updated the reference list as guided. |  |
| **Reviewer 1** |  |  |
| **Remarks** | **Authors Response** | **Reference** |
| The work seeks to access the HIV testing rate among unmarried young men in SSA. It was well written. However, there are some concerns that you need to address | We are grateful for your favourable comments. We would like to express our appreciation for your valuable expertise and the time you dedicated to our work. |  |
| **Question and remarks** | | |
| 1. The aim of the study was not well stated. | The study objective has been included in the abstract and introduction. |  |
| 2. Why was this study restricted to young unmarried men? Why did you exclude young unmarried women? Why did you also exclude married men and women? This was not explained in the manuscript. | Thank you for the guidance. We have included an explaination in the methods to explain our study sample.  The DHS dataset we used for the study contain only data of men aged 15-59 years (MR recode). Our decision to focus our study on umarried young women was informed by existing literature which shows that unmarried young men are more likely to engage in behaviors that put them at higher risk of HIV infection, such as multiple sexual partners, unprotected sex, or substance use. Assessing HIV testing rates among young men allows healthcare providers and policymakers to identify gaps in testing coverage and develop targeted interventions to increase testing rates.  Therefore, examining the HIV testing rates among unmarried young men is important for HIV prevention, vulnerability assessment, linkage to care, sexual and reproductive health services, and data-driven decision-making. |  |
| 3. How were the countries selected? Those excluded why were they excluded? | Sub-Saharan Africa has 48 countries. Of the 48 counties, 43 have conducted at least one DHS. Thirty-nine (39) had accessible DHS datasets. Of the 39 countries that had accessible DHS data, 19 conducted the most recent DHS between 2015 and 2022. Thus, all 19 countries with accessible DHS datasets conducted between 2015 and 2022 were included in our study. |  |
| 4. The discussion was poorly written. You did not relate it to other literatures on HIV testings among young unmarried men elsewhere. You did not give a possible reason for your findings and how it compared to findings elsewhere. | The discussion has been reworked to ensure that our findings have been discussed in comparison with what has been found in prior studies conducted in SSA countries |  |
| 5. Though, you mentioned in your limitation section that the data were collected at different years and may have been collected earlier than published, how did you ensure that despite this disparity, the data could be compared without bias? Are you sure the situation has not changed in some of the countries? | Thank you for this. Our understanding is that the DHS uses a standardised sampling design in all countries where the surveys are conducted. Therefore, if the data are collected from a similar population sub-group, defined DHS wave and analysis is weighted, it should be sufficient to compare the results. We also ensured that the datasets used were from the most recent surveys done in each country. We do acknowledge that the situation could have changed in the countries since the last DHS was conducted. However, there are no new measurements that have taken place due to the lack of new DHS data. Thus, the current DHS for these countries are the only available source of information to measure HIV prevalence since the censuses do not have this topic.  Thank you for the guidance. We appreciate your expertise. |  |
| **Reviewer 2** | We are grateful for your favourable comments. We would like to express our appreciation for your valuable expertise and the time you dedicated to our work. |  |
| The title does not read well and should be revised. | The title has been edited to read as follows **“Prevalence of HIV testing among the never-married young men (15-24) in sub-Saharan Africa: an analysis of Demographic and Health Survey data (2015-2022)”** |  |
| The third paragraph of the discussion needs to be revised. | The discussion section has been reworked after additional analysis was done |  |
| Some of the information presented in the introduction and discussion seems assumptive and needs to be substantiated with literature. Additional data collection (other data sets) and analysis would be helpful in deriving more sound conclusions and suggested changes to policy that would be both realistic and sustainable. | We have done multivariate regression analysis to strengthen the current analysis. This has helped us to identify determinants of the uptake of HIV testing among unmarried young men. The results have become more focused on informing recommendations for enhancing the uptake of HIV testing among young people |  |
| Acknowledgement of the range of data spanning a substantial time period (2010-2022) as a limitation was noteworthy but this does pose a problem to the accuracy of the analysis. | We have limited the analysis to countries with DHS data for the period 2015 -2022 |  |
| Perhaps the data should be limited to a smaller time window as advances in HIV diagnosis, research and therapeutics have occurred at a rapid rate within the past 20 years. | We have limited the analysis to countries with DHS data for the period 2015 -2022 |  |
| Furthermore, due to certain countries within SSA not have accessible DHS datasets this could possibly not be an accurate representation of SSA. | Thank you for the guidance. This has been edited appropriately since the countries have inaccessible data (Bostwana, ) all their DHSs were done before 2015. As such the said dataset was not relevant to this current study |  |
| This being said, the study does make use of good data analysis techniques and draws some important conclusions which may influence healthcare policies. Additional suggestions/recommendations could be helpful in this regard. | Thank you for the guidance. We appreciate your expertise. We have provided additional recommendations based on the additional analysis done. |  |
