# Supplementary material for: Prevalence of HIV testing uptake among the never-married young men (15–24) in sub-Saharan Africa: An analysis of demographic and health survey data (2015–2020)
Source: PLoS One. 2023 Oct 5;18(10):e0292182. doi: 10.1371/journal.pone.0292182 (PMC10553359; doi:10.1371/journal.pone.0292182)
Supplement: Attachment — Submitted filename: Rebuttal letter.docx [file pone.0292182.s004.docx]

The Editor,

PLOS ONE

12^th^ July 2023

**Sub: Response to Decision Letter: Manuscript ID: PONE-D-23-03270: Prevalence of HIV testing among the never-married young men (15-24) in sub-Saharan Africa: an analysis of Demographic and Health Survey data (2015-2022)**

Refer to the above subject matter.

We are grateful to the editor and reviewers for their time and constructive comments on our manuscript. We have taken into consideration the second round of reviews and suggestions from the editor, and we would like to submit a revised version of the manuscript for consideration by the journal. To reflect changes or amendments made in the revised version of the manuscript, changes or amendments have been highlighted in the current version of the manuscript. In addition, we provide a point-by-point response explaining how we have addressed the editor’s comments in the section following this one. We are looking forward to hearing the results of your evaluation.

Yours sincerely,

Lead author

Emmanuel Musonda

On behalf of co-authors

| **Journal Requirements** | | |
| --- | --- | --- |
| **Editors Remarks** | **Authors Response** | **Reference** |
| Thank you for submitting your manuscript to PLOS ONE. After careful consideration, we feel that it has merit but does not fully meet PLOS ONE’s publication criteria as it currently stands. Therefore, we invite you to submit a revised version of the manuscript that addresses the points raised during the review process. | We would like to extend our gratitude to the editor and reviewers for their valuable time and constructive feedback provided on our manuscript. |  |
| Please review your reference list to ensure that it is complete and correct. If you have cited papers that have been retracted, please include the rationale for doing so in the manuscript text, or remove these references and replace them with relevant current references. Any changes to the reference list should be mentioned in the rebuttal letter that accompanies your revised manuscript. If you need to cite a retracted article, indicate the article’s retracted status in the References list and also include a citation and full reference for the retraction notice. | We have updated the reference list to align with PLOS ONE’s recommended referencing style “Vancouver” with the first six authors et al.  We have also verified that none of the references we have cited has been retracted. | **Reference list** |
