# Supplementary material for: Prevalence of HIV testing uptake among the never-married young men (15–24) in sub-Saharan Africa: An analysis of demographic and health survey data (2015–2020)
Source: PLoS One. 2023 Oct 5;18(10):e0292182. doi: 10.1371/journal.pone.0292182 (PMC10553359; doi:10.1371/journal.pone.0292182)
Supplement: Attachment — Submitted filename: Reviewers Response.docx [file pone.0292182.s006.docx]

The Editor,

PLOS ONE

28^th^ August 2023

**Sub: Response to Decision Letter: Manuscript ID: PONE-D-23-03270: Prevalence of HIV testing among the never-married young men (15-24) in sub-Saharan Africa: an analysis of Demographic and Health Survey data (2015-2020)**

Refer to the above subject matter.

We are grateful to the editor and reviewers for their time and constructive comments on our manuscript. We have taken into consideration the second round of reviews and suggestions from the editor, and we would like to submit a revised version of the manuscript for consideration by the journal. To reflect changes or amendments made in the revised version of the manuscript, changes or amendments have been highlighted in the current version of the manuscript. In addition, we provide a point-by-point response explaining how we have addressed the editor’s comments in the section following this one. We are looking forward to hearing the results of your evaluation.

Yours sincerely,

Lead author

Emmanuel Musonda

On behalf of co-authors

| **Journal Requirements** | | |
| --- | --- | --- |
| **Editors Remarks** | **Authors Response** | **Reference** |
| Thank you for submitting your manuscript to PLOS ONE. After careful consideration, we feel that it has merit but does not fully meet PLOS ONE’s publication criteria as it currently stands. Therefore, we invite you to submit a revised version of the manuscript that addresses the points raised during the review process. | We would like to extend our gratitude to the editor and reviewers for their valuable time and constructive feedback provided on our manuscript. | The changes made to the manuscript are in highlighted in red. |
| 1. Reference(s) is/are need for your reasons to include only unmarried men | Thank you for the guidance. We have included references justifying our analysis sample group, that is unmarried men. | Line 120 |
| 2. Line 108: in your feedback to the reviewer, you indicted that a total of 19 countries were included however, in the manuscript you indicated 18 countries. this discrepancy need to be resolved. | Thank you for bringing this to our attention. The discrepancy has been resolved. The correct position is 18 countries as indicated in the manuscript. | Table 1 |
